# QSAR Study on Thiazolidine-2,4-dione Derivatives for Antihyperglycemic Activity

**DOI:** 10.4103/0250-474X.45392

**Published:** 2008

**Authors:** B. R. Prashantha Kumar, M. J. Nanjan

**Affiliations:** Department of Pharmaceutical Chemistry, JSS College of Pharmacy, Rocklands, Ootacamund-643 001, India; 1 TIFAC CORE, JSS College of Pharmacy, Rocklands, Ootacamund-643 001, India

**Keywords:** QSAR, Diabetes mellitus, thiazolidine-2, 4-diones, antihyperglycemic activity

## Abstract

A set of seventy four molecules belonging to the class of thioglitazones were subjected to the QSAR analysis for their antihyperglycemic activity. All the molecules were subjected to energy minimization to get 3D structures, followed by conformational analysis to get the conformation of the molecule associated with the least energy and highest stability. Various physico-chemical parameters were then calculated using ALCHEMY 2000 software, namely, thermodynamic parameters, structure-dependant parameters, topological parameters and charge-dependant parameters. Multiple linear regression analysis was carried out on all the molecules. The final equation was developed by choosing optimal combination of descriptors after removing the outliers. Cross validation was performed by leave one out method to arrive at the final QSAR model for the chosen set of molecules to exhibit antihyperglycemic activity.

Diabetes mellitus is a major health concern especially in the urban world. Studies showed that there are 150 million people suffering from diabetes mellitus and by 2025 it is estimated that the figure would rise to 300 million^1^. Over 90% of the diabetes mellitus patients are type-2 patients^2^. Type-2 diabetes mellitus is now considered as a life-style disease and is usually associated with urbanization, mechanization and change in life-style habits^3^. This disease is characterized by insulin resistance and cardiovascular dysmetabolic syndrome. The conventional therapy of type-2 diabetes mellitus (sulphonylureas) has not been satisfactory as it is not successful in treating associated cardiovascular risk factors, which is the major cause of morbidity. The current trend is, therefore, to make the therapy better by choosing appropriate combination of available drugs. A parallel search for newer drugs is also being made.

Thiazolidine-2,4-diones are the class of oral hypoglycemic agents which increase insulin sensitivity at target tissues like liver and skeletal muscles. In addition, it also improves the markers of cardiovascular risk factors by decreasing the free fatty acids and altering the lipoprotein metabolism. Thiazolidine-2,4-diones act on Peroxisome proliferator activating receptor-γ (PPAR-γ) receptors^4^ which regulate the gene expression mainly in the adipose tissues. PPAR-γ is a member of subfamily, which belongs to a 48 member nuclear receptor super family. After binding with thiazolidine-2,4-diones, a conformational change occurs in the receptor which leads to the binding of a co-activator protein^5^. Rosiglitazone and pioglitazone molecules from the class of thioglitazones available in the market are showing severe adverse effects. The development of drugs from this class of compounds through lead optimization or through sophisticated computer-aided drug design (CADD) techniques is, therefore, the current need of the hour. The present QSAR study on various thiazolidine-2,4-diones attempts to address this need by arriving at the physico-chemical properties required for high antihyperglycemic activity in the form of a mathematical equation, according to the Hansch type of analysis^6^–^9^. This study should, therefore, help in designing newer molecules with better antihyperglycemic activity.

## MATERIALS AND METHODS

The hypoglycemic activity and the plasma triglyceride lowering activity data for 74 molecules tested, using genetically obese and diabetic yellow KK mice, was taken from the literature as reported by Takashi *et al*^10^. The literature values and the general structure of the molecules are given in Tables 1–5. The compounds examined have been screened for their antihyperglycemic activity by the same procedure, to avoid incongruency of data. The reported hyperglycemic activities were originally assigned numbers from 1 to 3 based on the percentage reduction of blood glucose concentration. These data were then converted into log molar antihyperglycemic activity data (pHGA) by dividing the original values by their respective molecular weights and taking the logarithms, as it would give numerically larger values for the active compounds than those of the inactive compounds. The log molar plasma triglyceride lowering activity (pTLA) data was also calculated in a similar way and used as a descriptor. The plasma triglyceride lowering activity was considered as one of the descriptors based on the mechanism of action of thioglitazones. Thioglitazones are known to produce antihyperglycemic activity by inducing triglyceride lowering activity, thereby reducing obesity and increasing insulin sensitization.

**TABLE 1:** BIOLOGICAL ACTIVITY DATA AND CALCULATED VALUES FOR 5-[4-(2-PHENYLETHOXY) BENZYL]-1,3-THIAZOLIDINE-2,4-DIONE DERIVATIVES 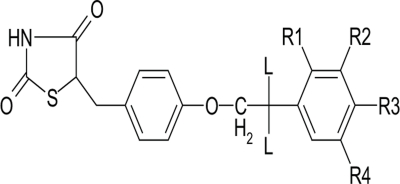

C.No.	R_1_	R_2_	R_3_	R_4_	L	HGA	Molar HGA	pHGA	pTLA	pHGA_c_	pHGA_e_-pHGA_c_
1.	H	H	H	H	CH_3_	3	8.451	-2.073	-2.073	-2.047	-0.025
2.	H	H	CH_3_	H	CH_3_	2	5.420	-2.266	-2.266	-	-
3.	OCH_3_	H	H	H	CH_3_	3	7.792	-2.108	-2.284	-2.063	-0.045
4.	H	OCH_3_	H	H	CH_3_	3	7.792	-2.108	-2.284	-2.098	-0.01
5.	H	H	OCH_3_	H	CH_3_	1	2.597	-2.585	-2.284	-	-
6.	H	H	C_2_H_5_	H	CH_3_	3	7.833	-2.106	-2.583	-2.220	0.114
7.	H	H	OC_2_H_5_	H	CH_3_	3	7.519	-2.124	-2.124	-2.079	-0.045
8.	H	H	H	OH	CH_3_	1	2.695	-2.569	-2.092	-	-
9.	H	H	H	H	H	3	9.174	-2.037	-1.912	-2.028	-0.009
10.	H	H	CH_3_	H	H	3	8.798	-2.056	-2.092	-2.101	0.045
11.	OCH_3_	H	H	H	H	3	8.403	-2.075	-1.951	-1.955	-0.120
12.	H	H	OCH_3_	H	H	3	8.403	-2.075	-2.075	-2.017	-0.058
13.	H	H	C_2_H_5_	H	H	3	8.451	-2.073	-2.270	-2.104	0.031
14.	H	H	OC_2_H_5_	H	H	3	8.086	-2.092	-1.967	-2.012	-0.080
15.	H	H	CI	H	H	3	8.310	-2.080	-2.080	-2.116	0.036
16.	OCH_3_	H	CH_3_	H	H	3	8.086	-2.092	-2.569	-2.132	0.039
17.	H	OCH_3_	OCH_3_	H	H	3	7.752	-2.110	-1.986	-2.083	-0.027
18.	H	OCH_3_	OCH_3_	OCH_3_	H	3	7.194	-2.143	-2.319	-2.088	-0.055
19.	H	-OCH_2_O-		H	H	3	8.086	-2.092	-2.092	-2.041	-0.051

c (Calculated activity), e (experimental activity). Molecules and their structures considered for the 2D-QSAR study along with experimental, calculated and residual activities.

**TABLE 2:**
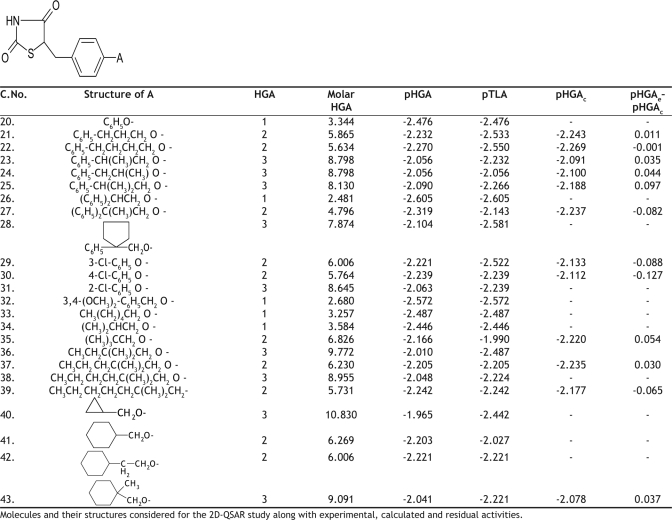
BIOLOGICAL ACTIVITY AND CALCULATED ACTIVITY DATA OF 5-BENZYL-1,3-THIAZOLIDINE-2,4-DIONE DERIVATIVES

**TABLE 3:**
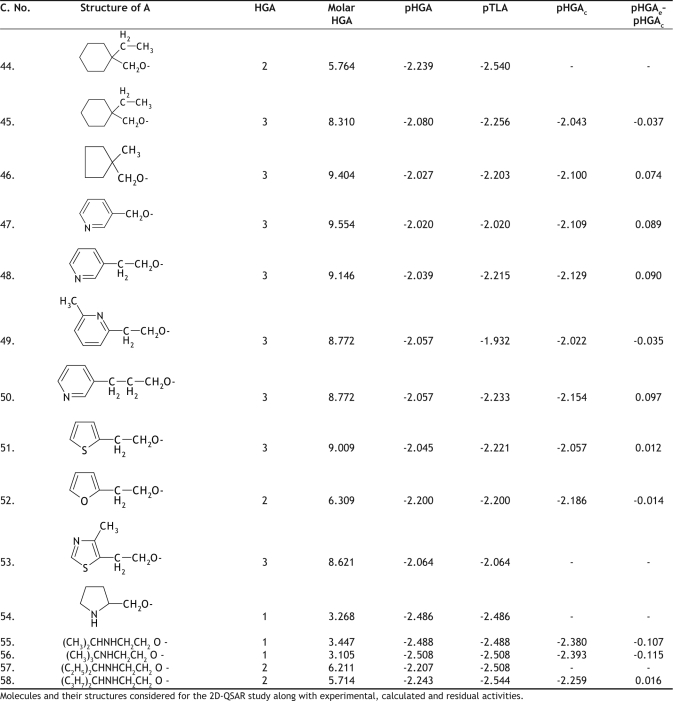
BIOLOGICAL ACTIVITY AND CALCULATED ACTIVITY DATA OF 5-BENZYL-1,3-THIAZOLIDINE-2,4-DIONE DERIVATIVES

**TABLE 4:**
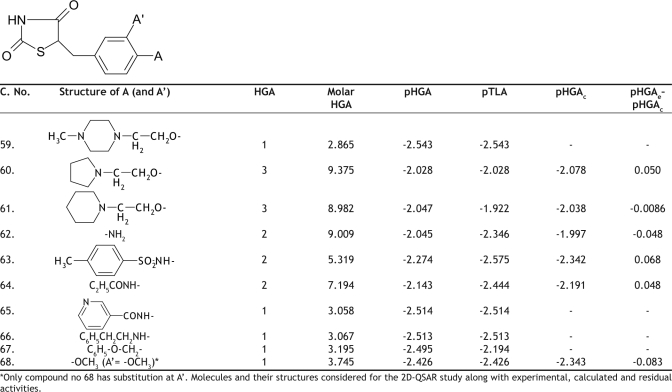
BIOLOGICAL ACTIVITY AND CALCULATED ACTIVITY DATA OF 5-BENZYL-1,3-THIAZOLIDINE-2,4-DIONE DERIVATIVES

**TABLE 5:**
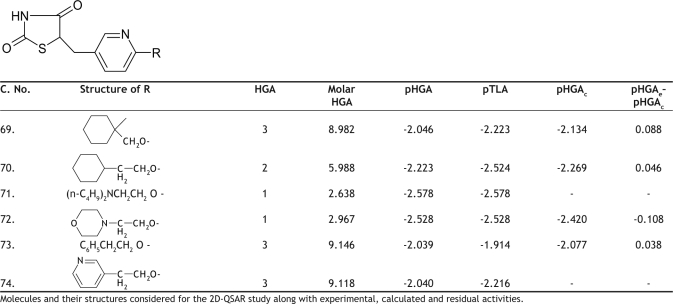
BIOLOGICAL ACTIVITY AND CALCULATED ACTIVITY DATA OF 5-(PYRIDINE-3-YLMETHYL)-1,3-THIAZOLIDINE-2,4-DIONE DERIVATIVES

All the 74 molecules were sketched using ALCHEMY 2000 (TRIPOS, USA)^11^ software and were subjected to energy minimization by using molecular orbital pac (MOPAC) method^12^. MOPAC calculations are semi-empirical calculations based upon a quantum-mechanical approach, used to optimize the geometry of a molecule depending on the charges on its various atoms. The pHGA was taken as the biological activity variable and the pTLA was considered as an experimental descriptor in the QSAR analysis. The triglyceride lowering action of thiazolidinediones is known to be the main cause of hypoglycemic action and, therefore, should show a strong correlation.

Thermodynamic, structure dependent, topological and charge dependent parameters were calculated for each energy minimized molecule using molecular mechanical methods of Sybyl force field^13^. The thermodynamic parameter used was octanol:water partition coefficient (LogP), which plays an important role in drug absorption. Structure dependent parameter, molecular polarizability (polar), was calculated by using atomic hybrid polarizability based on the additive approach given by Miller^14^. Specific polarizability (sp pol) is the ratio of polar/volume of a molecule. Molecular weight (mweight) and the volume (vol) which is the molecular volume of the molecule computed by the grid method of Boder were used^15^. Topological parameters like, molecular connectivity (Ka3)^16^, molecular connectivity indices (XV_0_), first order valance molecular connectivity indices (VX_1_), first order molecular connectivity indices (X_1_), third order molecular connectivity indices(X_3_), obtained from hydrogen suppressed graphs of the molecules and were calculated according to the method of Kier and Hall^17^,^18^. Third order molecular shape indices (Ka_3_), which encodes an atom's identity involved in assessing the shape of a molecule and Weiner indices (WeinI) were used. Charge dependent parameters like the sum of absolute charges on each atom of the molecule (ABSQ), the sum of charges on nitrogen and oxygen in the molecule (ABSQon), dipole (μ) computed based on the 3D structure and charges calculated by Gasteriger-Marsili method implemented in SciQSAR software and expressed in debye, largest negative charge over the atoms in the molecule (Maxneg) and largest positive charge over the atoms in the molecule (Maxpos) were also calculated and used for the QSAR study.

All the atoms were initially assigned a charge zero when calculating charges by the Gastiger-Marsili method. Through a reiterative routine, this method shifts charges from a less electronegative to a more electronegative atom, using the damping factor whose value is distance dependant. The latter prevents equalization of charges among the atoms. The final charge depends only on the nature of atoms and their connectivity to other atoms and not on the 3D structure of the molecule. All charges are expressed as a fraction of the electron charge.

## RESULTS AND DISCUSSION

Multiple linear regression analysis and other statistical analysis were carried out on all the 74 molecules. The outlier molecules were then removed to improve the equation's predictive power. The final set of equations was obtained using 50 molecules and the best equation was obtained by using the optimal combination of descriptors. Descriptors were selected for the final equation based on their correlation coefficients and those descriptors having intercorrelation coefficient below 0.7 were considered, to select the best equation. Cross validation by leave one out method was carried out on these final set of 50 molecules to further enhance and validate the predictive power of the equation. Acceptability of the regression equation was judged by examining the statistical parameters.

Various equations were obtained after performing multiple linear regression (MLR) Analysis. Equation predictive power was judged based on various statistical parameters like correlation coefficient (r^2^), Fischer statistical value (F) at the probability of zero and root mean square deviation (RMSD). All these statistical parameters are computed as defined in the ALCHEMY 2000 software.

**Fig. 1:**
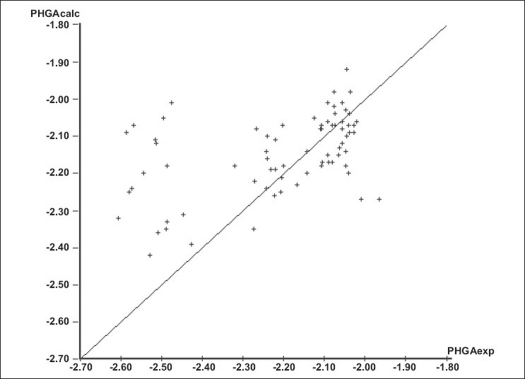
pHGA_calc_ versus pHGA_exp_ plot for regression analysis on initial 74 molecules. Multiple linear regression plot indicating the presence of outliers which are not correlating with experimental and calculated antihyperglycemic activity

The initial regression analysis was performed on all the 74 molecules which resulted in regression equation with poor predictive power (Table 6, Equation 1 ). The plot of pHGA_calc_ versus pHGA_exp_ for this equation is given in fig. 1. The plot indicated the presence of many outliers and that could be a possible reason for the poor predictive power. Molecules which affected the equation adversely were, therefore, considered as outliers and were removed to enhance the predictive power. The calculated antihyperglycemic activities of the 24 outlier compounds are not reported in Tables 1–5. Multiple linear regression equations developed for all the 74 molecules and the final set of 50 compounds along with the statistical parameters are given in Table 6.

**TABLE 6:** REGRESSION EQUATIONS OBTAINED BY DOING SEQUENTIAL MLR ANALYSIS

Eq. No.	Equation	n*	r^2^	F-value	RMSD
1.	PHGA = 6.41 + 0.47_P_TLA + 0.22X_1_ + 0.50VX_0_ + 0.27VX_1_ + 0.23LogP +6.77e-004WienI − 0.0719X_3_ + 5.86e-003Dipole − 0.27Polar − 1.55e-003Volume − 8.91e-003MWeight − 0.14ABSQ − 0.59ABSQon − 0.14MaxQp − 0.0548Ka_3_ − 4.43MaxNeg + 47.11SpPol	74	0.13	23.09	0.13
2.	PHGA = 25.81 + 0.32_P_TLA + 0.36X_1_ + 0.57VX_0_ + 0.24VX_1_ + 0.10LogP + 1.78e-005WienI + 0.17X_3_ + 0.0516Dipole − 0.66Polar + 0.0465Volume − 0.0181MWeight − 0.15ABSQ - 0.23ABSQon -0.65MaxQp + 0.10Ka_3_ − 4.52MaxNeg + 1.94eSpPol	50	0.84	20.18	0.05
3.	PHGA = 22.99 + 0.31_P_TLA + 0.13LogP + 0.0776Dipole − 0.57Polar + 0.0704Volume + 0.0389ABSQon − 0.12Ka_3_ + 1.73eSpPol	50	0.82	42.78	0.06
4.	PHGA = 22.57 + 0.30_P_TLA + 0.14LogP + 0.0810Dipole − 0.55Polar + 0.0685Volume + 0.0485ABSQon − 0.13Ka_3_ + 1.70eSpPol	49	0.83	43.59	0.06
5.	PHGA = 23.42 + 0.33_P_TLA + 0.18LogP + 0.0683Dipole −0.61Polar +0.0754Volume − 0.14ABSQon − 0.14Ka_3_ + 1.78eSpPol	49	0.86	44.26	0.05

* n = Number of molecules on which regression analysis was performed. Multiple linear regression equations developed along with the statistical parameters for all the 74 thioglitazones considered for the QSAR study and for the final set of 50 thioglitazones.

**Fig. 2:**
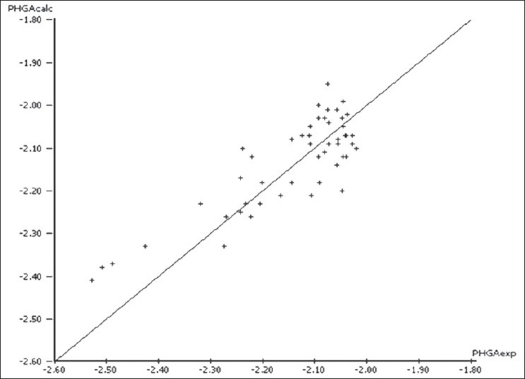
pHGA_calc_ versus pHGA_exp_ plot for regression analysis on 50 molecules. The multiple linear regression plot obtained after the removal of 24 outliers depicting a better correlation between experimental and calculated antihyperglycemic activity.

The final regression analysis was performed on a set of 50 molecules after exclusion of the outliers from the original training set. Removing the outliers considerably increased the predictive power of the equation as seen in Eqn. 2. Eqn. 2 showed a good regression coefficient but did not show a favorable F-statistical value. This may be due to the self fitting of the regression equation as more number of descriptors was used. To increase the F-statistical value, the descriptors which did not affect the regression equation were sequentially removed. Eqn. 3 was thus obtained by doing so. The pHGA_calc_ versus pHGA_exp_ plot for this equation is shown in fig. 2. However, there was no further improvement in the predictive power of the equation despite removing related parameters which in turn indicated the importance of their presence in the model.

Cross validation was performed using leave one out method (LOO) on these final 50 molecules. Cross validation process eliminates one compound from regression and predicts the activity for the rvemaining molecules in the set. The best QSAR model developed out of the 50 molecules was equation 5. This equation was obtained for 49 molecules, after leaving compound 30. The pHGA_calc_ values for the final set of 49 molecules according to the final equation (Equation 5) are given in Tables 1–5. The Eqn. 4 obtained after removing the compound 11 was also impressive but not good when compared to Eqn. 5 in terms of statistics.

The best QSAR Eqn. 5 indicates that triglyceride lowering activity is one of the important descriptors. It is also well known experimentally that reduction of triglyceride levels leads to reduction in blood glucose levels. The LogP value (partition coefficient), which plays an important role in the passage of the drug across the phospolipid bilayer during absorption, is also playing an important role in exhibiting antihyperglycemic activity of the titled compounds. Parameters such as dipole moment (which depends on the presence of polar bonds), molecular polarisability or specific polarisability (which in turn depends on the polarity of the molecule), Volume (which plays an important physical role in binding to the receptor and in this case the PPAR-γ) and Third order molecular shape indices (which encodes an atom's identity involved in assessing the shape of a molecule towards the desired target) also play important role. The sum of charges on nitrogen and oxygen atoms in the molecule (ABSQon) also seems to be quite important in terms of enhancement of the electron density due to the higher electronegativity of these atoms.

The developed QSAR model can be utilized for the further development of new molecules belonging to the class of thioglitazones to exhibit good antihyperglycemic activity, as it reveals the various physico-chemical parameters that play important roles in exhibiting potential antihyperglycemic activity. Work in this direction is in progress and will be reported in our next publication. Based on the developed QSAR model, it may be concluded that partition coefficient, polarity, polarisability, volume, number of nitrogen and oxygen atoms in relation to their charges and the molecular shape of the molecule are the properties that are to be considered apart from triglyceride lowering activity (experimental parameter), while designing newer thioglitazones, for their potential antihyperglycemic activity.
